# Hospital and Institutional News

**Published:** 1912-02-17

**Authors:** 


### HOSPITAL AND INSTITUTIONAL NEWS.

### LORD LISTER'S PEERAGE.

We deal elsewhere with humanity's debt to Lord Lister and with the well-known revolution in surgery which the aseptic method has brought about. Of the unscientific aspects of his career, however, it may not be uninteresting to speak. Lister, who had been created a baronet in 1883, was raised to the peerage at the Diamond Jubilee of 1897, and, as the hospital world is probably aware, this is the first peerage ever conferred upon a surgeon. It is also only the second which has honoured scientific distinction, Lord Kelvin (Sir W. Thompson) being the first. Of Lord Lister's other honours we will recall only that he was, with Florence Nightingale, one of the original members of the Order of Merit, and from 1895 to 1900 President of the Royal Society. It is a fact perhaps worth recording that the offer, on condition of cremation, of burial in the Abbey of recent years has been almost invariably frustrated by the private wishes of the dead, while in those cases for which the public first expressed a desire to honour some great man, Meredith for instance, the Dean, if we remember, did not see his way to accede to the request. It is, however, satisfactory to note that Westminster Abbey is now making cremation a condition of burial in its precincts, not only for the sake of the Abbey, but also on behalf of the classic and most hygienic form of burial.

### FIRST HONOURS FOR LISTER.

Up to so recently as 1882 Lister's work was practically unrecognised, though its effects were clearly shown by statistics which were unassailable. This was proved by the paper read before a large and representative audience by Sir Henry Burdett in September 1882, entitled "The Relative Mortality after Amputation of Large and Small Hospitals" (Churchill). In this paper Sir Henry showed the enormous effect which Lister's work and that of Spencer Wells had had on the saving of human life. In quoting the figures and results of both these great surgeons, Sir Henry said: "How has it happened that two such men as Lister and Wells, whose names are no small glory to England as benefactors to humanity at large, have not ere this received the highest honour which Government ever bestows upon medical men? Is it because fashion or custom, or both, have decreed that Courts and Governments should confer the highest honours on those who are most successful in destroying life on a large scale, and not on those who save life? No wonder if the flower of our university youth choose the Church, or the law, the army or navy, or some branch of the civil service of the State, rather than the medical profession, because they at once take an enviable social position, and a successful career may lead to titles and pensions, and to a seat in the House of Lords. It has been well asked why should a baronetcy be the highest titular distinction conferred upon members of the medical profession? Is Jenner or Paget less worthy of a life peerage than the eminent men who now sit on the bench of bishops, or any of the lawyers, soldiers or sailors who have been rewarded by hereditary peerages? Can any member of the House of Lords do greater service to his country in that assembly than an eminent member of the medical profession could render in the promotion of legislation for securing and protecting the public health?" Sir Henry's paper had a wide circulation, and called forth considerable interest. Apart from its immediate results—both Spencer Wells and Joseph Lister were made baronets within a few weeks—the quotation in the circumstances is of permanent historical value.

### LORD LISTER AND THE KING'S FUND.

Lord Lister always displayed the deepest interest in promoting the welfare and efficiency of the voluntary hospitals. He became a member of the General Council of the King's Fund on its establishment in 1897, was appointed chairman of its Distribution Committee, and worked energetically in this capacity from 1898 to 1903 inclusive, and was even a member of the Visiting Committee during the same six years. Those who had the pleasure of serving with Lord Lister on the Distribution Committee are not likely to forget the thoroughness with which he entered into every question, as it arose, and his strict impartiality. As a colleague he was always hospitable, and once he formed a friendship it endured throughout life. Few men of his ability and character have succeeded, as Lister did, in making it a principle of their daily intercourse to overcome irritation, and to meet and discharge the duties of life without friction, and with invariable courtesy, which had a special charm. The writer can testify on all these matters from a close and continuous intercourse during a period of Lister's life when he was called upon to handle and decide, in trying circumstances, problems of infinite difficulty.

### THE NEW MEDICAL DIRECTOR OF THE ANTI-PHTHISIS CAMPAIGN.

We understand that Mr. Marcus Paterson, medical superintendent of the Brompton Hospital Sanatorium at Frimley, Surrey, is leaving in order to take over the post of Medical Director to the Welsh National Memorial, and to assist in the working out of this scheme for the prevention and abolition of tuberculosis in Wales. Mr. Marcus Paterson, who is an M.B., B.S. of Durham, and who studied at Newcastle-on-Tyne and S. Mary's Hospital, not merely has had wide practical experience in the administration and treatment of phthisical cases, but has contributed important publications to the literature of the subject. Thus his "Auto-intoxication in Pulmonary Tuberculosis" and his "Suggestions for the Future of Consumptive Patients of the Working-classes," which latter appeared in the *Lancet*, together with his "Graduated Labour in the Treatment of Pulmonary Tuberculosis" indicate pretty clearly the amount of his work and the lines of his thought upon this great question. It may be asserted confidently that the Welsh Memorial Committee could have found no one better fitted by practical experience and originality of thought to give that unity and direction without which the best intentions and the largest supply of money can run only to waste. Readers of The Hospital more than once have had the opportunity of testing this assertion from reading Mr. Paterson's signed articles in our columns.

### HASTINGS AND ROCHDALE COMPETITIONS.

The results are published of two architectural competitions for hospital plans at Hastings and at Rochdale. At the former Mr. E. T. Hall, F.R.I.B.A., the assessor in the East Sussex Hospital competition, has given his award as follows: First, Messrs. J. S. Snell and S. M. Spoor (conjointly); second, Messrs. C. K. and T. C. Mayor; third, Messrs. Adams and Holden. The designs were publicly exhibited in the Drill Hall, Middle Street, Hastings, from February 13 to 17 inclusive. As noted in our issue of September 9, 1911, the total sum available for the completed scheme is £35,000. At Rochdale the result of the competition, limited to local architects, for Infirmary extension designs is announced, and the chosen plans are those by Mr. Hugh Healey, A.R.I.B.A. Premiums of 50, 30, and 20 guineas were offered, and eleven sets of plans were submitted.

### A NOTABLE HOSPITAL WORKER.

The late Alderman G. J. Johnson of Birmingham was one of a small group of men who in the early sixties began voluntarily to give much time and thought to the maintenance of the voluntary system of hospitals. As a solicitor in large practice he was naturally concerned with the constitution of these institutions. He took great pains and devoted much time to the perfecting of their by-laws and regulations, and was associated with the late Mr. Sampson Gamgee in the successful efforts first made to attract the support of the working classes to the voluntary hospitals. These efforts eventuated in the Hospital Saturday collections in Birmingham, which have flourished so abundantly there. Mr. Johnson was never too busy to give time and thought to hospital work, and he supported every improvement and change of a practical nature. Many of these changes have helped to create the modern hospital as it stands to-day. Personally he was one of the ablest, most pleasant, and knowledgeable men we ever met, and such a staunch friend. His death will cause a gap in the life of many friends who survive him, which can never be filled. Birmingham owes many good things directly or indirectly to the late Mr. Johnson, who was an intimate associate of the late C. E. Matthews, J. G. Bunce, and other Birmingham worthies who predeceased him.

### THE ALMONER AS THE CENTRE OF HOSPITAL WORK.

Miss Cummins, Lady Almoner at St. Thomas's Hospital, took the opportunity afforded her by the meeting last week of the Acton Central Aid Society to deliver a capital address on "Co-operation between Hospital Almoners and Relief Societies." In the course of it she said Mr. Loch, who had been inquiring into the matter, saw that hospital treatment was often stunted in its good effects by the lack of right conditions under which the treatment recommended had to be carried out, and if the hospital could have attached to it a consulting-office to act as a "clearing house" much of the distress would be alleviated. Seven years ago St. Thomas's had instituted an almoners' department, over which she had ever since presided, and a complete scheme of social service was carried on, with the aid of outside voluntary workers and various charities. There were, in almonership, two distinct areas of work: relief work and friendly visiting. Co-operation between the hospital and outside charity consisted of two kinds: material relief, and personal service of kindly visits, which did so much to help and cheer. It was particularly necessary that a hospital should not be considered another relieving office. Hospital almoners' work could not be carried on with one-fifth of its present success were it not for the co-operation of charity workers and societies. At the hospital they often needed to know all that could be known about a case, the environment and condition of home life, etc. Then they sent a letter to an outside "visitor," and asked for his or her impressions, and we are glad to note, the hospital visitor was generally a welcomed guest.

### NEW APPOINTMENTS AT LIVERPOOL.

Mr. Ralph Brocklebank, the newly elected President of the Liverpool Royal Infirmary for the ensuing year, succeeds Sir Archibald Williamson, M.P., at a very interesting time in the life of the institution. Last March, it should be recalled, the new out-patient department was opened, when some notable persons were on the platform, and last August the strike riot, as our readers will remember, taxed the hospital's administration to the utmost. But apart from this, Mr. Brocklebank's retirement, after thirty years as Treasurer and then five years Chairman of the Committee, has removed, from routine work at least, one of the most enthusiastic workers for the hospitals in recent times. The Board has also lost the services of Mr. J. L. Birkett, who has been a member for forty-three years.

### A NEW DEVELOPMENT.

Negotiations are proceeding between the Royal Infirmary and the Liverpool School of Tropical Medicine to place some wards at the school's disposal, for the benefit of University students who require clinical material close at hand. The Royal Infirmary's accounts show a loss of £160, for which Hospital Sunday in Liverpool is largely responsible. The fact that subscriptions have gone up is enough to show beyond the possibility of misconception that the congregations have produced a smaller total merely because the clergy have come to care about Hospital Sunday and its claims less and less. As Sir Archibald Williamson said in another connection: "It is individual effort which helps to bring money to an institution." What the want of it can achieve the clergy should know better than anyone. Sir Archibald Williamson took the opportunity given by the annual meeting to make a capital speech showing the anxiety caused by the Insurance Act, which, he said, would probably increase the work and so make each hospital require as much, if not more, support than before. He uttered also a weighty protest against State or rate-supported institutions.

### AN INTERESTING GIFT TO THE TAUNTON HOSPITAL.

Another example of the interest in local hospitals which families take and preserve all over England is the gift of £1,000 from the Hon. Mrs. Portman to the Taunton and Somerset Hospital in memory of her late husband, the Hon. E. W. B. Portman. At the time of his death, in April last, The Hospital published a memoir of his services to the institution of which he was President, so this gift, it will be seen, has a special significance and value. Moreover, the hospital's centenary is this year, and as we have before pointed out, the proposal is to celebrate it by raising an endowment fund of £30,000.

### A SUCCESSFUL IRISH HOSPITAL.

The South Infirmary, Cork, was very lucky last week in having an annual meeting not merely concerned with an exceptionally interesting year's work—the Insurance Act's sudden appearance alone will make 1911 remarkable in the annals of anxiety —but for having speakers able to make the most of their opportunity. We note that the new extern medical department has been successful enough to create the demand for "a similar addition to the extern surgical department." Mr. James F. M'Mullen, the honorary architect, whose work for the South Infirmary has been recorded more than once in these columns, was responsible for the new building. Last June initiated a new interest by the practical demonstration given by the city fire brigade with life-saving apparatus, and at Captain Hutson's suggestion new appliances and increased hose-lengths have been added to the hospital. The Most Rev. Dr. Browne made a good speech on the Insurance Act, and the chances of its amendment, and gave high praise to the House Committee and to Mr. S. H. Newsom, the hon. secretary and treasurer, who is as much to the fore in his own institutional work as he showed himself to be at the time of the conference held in Cork of the local hospitals to consider the Insurance Bill—as it then was.

### THE KING EDWARD MEMORIAL AT BRISTOL.

We are glad to learn from the speech of Sir George White, at the annual meeting of the Bristol Royal Infirmary, that the structure of the new King Edward Memorial wing is nearing completion. The building, we understand, will form three sides of a square, having in the centre an open space to be used as a garden for patients. £35,000 is still required, one half of the total cost. The need for further efforts to secure this is apparent from the fact that there was a deficit of over £5,000 on the year's work. There were also increases of in and out patients, 563 more of the former and 3,627 more of the latter having been treated.

### THE FINE ART OF MILK ADULTERATION.

For some time two or three preparations have been extensively vended to milk retailers, the main virtue of which has been advertised that they cannot be detected by analysis in samples of milk to which they have been added. Such a boast should of itself in any well organised community lead at once to the condign punishment of the vendor; but the Local Government Board presumably feels that the law is not strong enough for such a common-sense remedy and prefers rather to put analysts on their guard by publishing a full exposure of the mystery which the adulterators affect. The most extensively used and unblushingly advertised of these milk preservatives is one marketed under the name of "mystin." The Board's inspector has analysed samples of mystin and reports that they consist of sodium nitrite, formaldehyde, and oil of peppermint. The proportions of milk and mystin recommended for admixture by the ingenious proprietors of this concoction are such that the consumer would imbibe two grains of sodium nitrite in a quart of milk, and a considerably smaller dose of formaldehyde. Now such a dose of sodium nitrite cannot possibly be regarded as a negligible quantity, and it is, in fact, quite sufficient to cause very serious symptoms in certain cases of no very uncommon occurrence. So that the sale and use of mystin undoubtedly constitute a menace to the public safety, and should be (as we trust they are) a punishable misdemeanour.

### THE EXPLANATION.

Now the Local Government Board report further remarks that sodium nitrite is not a preservative, so that at first glance it might seem mere stupidity of the adulterator to use it. But the explanation is that sodium nitrite interferes with the ordinary tests for the presence of formaldehyde, and is thus introduced as a refinement in the art of adulteration for the express purpose of baffling the public health officials. Such misplaced ingenuity would surely find a profitable outlet in some less questionable application; at all events, now that the mystin bubble is pricked it may be hoped that the same clever chemist who evolved the idea of it will not continue to devote his talents to the disservice of invalids and children. The havoc that may be (possibly has been) wrought in hospitals through a milk supply containing mystin is almost too serious for contemplation. Fortunately any hospital dispenser could apply the tests for sodium nitrite if he were asked to do so; and now that attention has been called to the matter it may be hoped that the sale of this pernicious mixture has received a heavy set-back.

### THE WORK OF THE LONDON FEVER HOSPITAL.

The Right Hon. Lord Balfour of Burleigh took the chair at the annual meeting of the London Fever Hospital in Islington on Thursday last week. Dr.

William Hunter, senior physician to the hospital, read a report which was on this occasion rather more extensive than usual. It was made the vehicle for the production of some new statistics by Dr. Hunter, showing the proportion of the notified cases of scarlet fever and diphtheria over and above those admitted to the hospitals of the Metropolitan Asylums Board. Of these cases an increasing percentage is being admitted into the London Fever Hospital, where the more private accommodation and the facilities for isolation available continue to appeal to the class of patient for whom this hospital is intended. As regards the considerable epidemics of measles and rubella (German measles) which occurred in London in the early part of last year no fewer than some 600 patients were admitted. The admissions for 1911 were thus raised to nearly a record for the history of the hospital, which, we may remind our readers, extends over some 110 years. Lord Balfour of Burleigh emphasised the urgent necessity for more isolation accommodation and for more private rooms, which are always in demand, and appealed for funds to convert a disused wing into a number of small isolation rooms. The Secretary, Major Christie, was able to report that several legacies and donations had been received recently, which would help to neutralise a considerable falling off in legacies which occurred in 1911, and it is hoped that a beginning may be made towards extending the number of private rooms which make it a unique institution in London. Among the changes in the staff we may note the retirement after 27 years' service of Dr. Sidney Phillips from active duty and his election to the consulting staff; the promotion of Dr. Charles Box to the post of full physician, and the election of Dr. Jewesbury, assistant physician at Charing Cross Hospital, to the vacancy of assistant physician to the hospital in the place of Dr. Box.

### A HOSPITAL SHIP FOR THE NAVY.

The Medical Director-General of the Navy, Sir James Porter, took the opportunity provided by his presence at the dinner of the West London Medico-Chirurgical Society to announce that in eighteen months the first ship built for hospital purposes would be ready for the use of the Navy. He claimed this rightly as a triumph for the medical profession, which at last had enforced its view of the necessity for such a vessel. It is, however, something of an exaggeration to describe the new ship as "the first hospital ship in the world," for though it is, no doubt, the first Government hospital established as a permanent department in time of peace, the South African War produced many, the *Maine* among them, which voluntary agencies supplied for the use of the wounded. As usual, the Americans are carefully marking time before venturing to copy Great Britain's enterprise and example.

### THE NEW DEVONSHIRE SPA.

In view of the great interest excited by our recent series of descriptive articles on the spas of England, it is interesting to note that Torquay is to be turned into a spa shortly. Mr. A. J. Taylor, of Bath, is advising the Corporation, which has decided to spend £13,000 on medical baths and saloons. We understand that a promenade with a sea view, 29 dressing-rooms, and 13 bathrooms will be installed.

### UNJUSTIFIABLE OPERATIONS AGAIN.

In the February number of *Public Health* is reported the case of a labourer summoned for cruelty to children by refusing to allow his five-year-old daughter to be operated on for cleft palate. A small fine was imposed and the application granted of the National Society for the Prevention of Cruelty to Children for custody of the child for six months in order that the operation might be performed. Here we have brought up again pretty clearly the question of justifiability of compulsion to undergo a surgical operation; although, first of all, one or two side issues have to be disposed of. The man was admitted to be quite respectable, and, save in this particular instance, a good father. Then it might be added that although the prosecution claimed that without operation the child's speech would be considerably affected, yet, as a matter of fact, results are not very good as regards this. Further, as Mr. Bland Sutton has shown, the condition of cleft palate is often hereditary, as, too, according to less known but not less considerable writers, the general tendency to bodily malformations. The wisdom of expending time, skill, and money upon so—eugenically speaking—unworthy a member of society thus becomes debatable. Finally, we might note that one or other side seems to have missed a point in tactics by not making a good deal of the answer to Uncle Toby's pithy query —"And what said the mother to it?" However, these are of course accidents of the particular case. The question here suggested is—granted thorough justification for an operation, should that operation be enforced upon an individual who objects to undergo it?

### THE QUESTION OF COMPULSION.

Upon the larger issues involved, the issues of duty, of ethics, of parental rights, we have, of course, nothing to say: a matter of less consequence because the views taken upon these great points change from age to age, and indeed, as becomes noticeable nowadays, from decade to decade. But something may be mentioned as to considered medical opinion upon such cases. Not that that opinion is at all uniform. It is a matter of common observation that inborn temperament defies pretty often the modifying influence of scientific training. There are doctors whose disposition is, to import (why have we so few verbal exports?) a convenient French word, highly *autoritaire*. There are also doctors whose sympathies are always with revolution. Had the first-named doctors lived shortly after the time of Galileo, they would have spoken of him as a tactless fellow the obtrusion of whose convictions necessarily got him into trouble; while the latter, although convinced that vaccination keeps off smallpox, are seized (to use the latest expression) with *angst* at the idea of forcing the most ignorant clodhopper or the most ranting Little

Bethelite to allow his offspring's arm to be scratched with a needle. Against compulsion in a matter of life or death probably no medical man could be found.

### A NEW ROLE FOR THE HOSPITAL SECRETARY.

Out of this dilemma, of course, the only way is that old and famous, yet inglorious, mean path between the two extremes. Let the anarchist meet the *autoritaire* half-way. But one novel suggestion might be made. Hospital lay officials, such as committee men and secretaries, are, we think, especially likely to be able to furnish a valuable opinion in particular cases of this sort. They are familiar with the facts as to what surgeons can do; they are good gaugers of lay opinion on medical matters; and they are men of affairs used to conciliating people. If some way could be found of utilising their services in a sort of preliminary tribunal over these matters, the result might be often the saving of legal proceedings, and always a slow but certain educating of public opinion.

### A BEQUEST TO A HOSPITAL REFUSED.

The action which the above words imply is rare enough to be worth recording. The institution concerned is the Manchester Eye Hospital, which refused a legacy of £130 from Mrs. Rose Hyland because of the condition which she attached that "within twelve months time two lady members should be appointed to the Board of Management." "In view of our system of government," Mr. P. W. Kessler is reported to have said, "by a board of management of business men, by a ladies' committee which attends to the interior work of the hospital, and by a medical board, the management unanimously decided to make no alteration." It is also stated that the Ladies' Committee concurs in this view. At the Bedford County Hospital in May last year, The Hospital drew attention to the good results that had been obtained by the presence of two ladies on the Board, when a third lady offered herself for election. The third lady did not succeed, as it happened, but her right of candidature was admitted. Local conditions no doubt require local modifications, but though a legacy of £130, even to an institution like the Manchester Eye Hospital with a last year's overdraft of £544, may be too small to induce a change of constitution, we believe that ladies on committees of management where elected have not proved unsuccessful.

### THE GERMAN HOSPITAL AND ENGLISH PATIENTS.

Baron Bruno von Schröder, the treasurer of the German Hospital, Dalston, E., presided over his hospital's recent annual meeting, one of the interesting features of which was that part of the report, presented by Mr. W. F. Cochrane, the secretary, which gave the proportions of different nationalities among the 1,838 patients received. At the ordinary hospital the nationalities, for instance at the Seamen's Hospital, are varied enough, but at an institution like the German or the French the variety has rather a different air. About 470 were English (174 being accidents) and 420 were of the Jewish religion, enough to show that Mr. Cochrane need not, and we hope does not, rely too exclusively on the support of the German colony.

### A HOSPITAL AND ANONYMOUS LETTERS.

Encouraged doubtless by the newspaper which published the letters, anonymous writers have been charging the Liverpool Dental Hospital with abuse. From the somewhat confused speeches which were made at the annual meeting it would seem that food for misconception in the public mind has been found in the debt of £4,000 on the building fund, and in the relation of the hospital to its school, where the education of dental surgeons in mechanical dentistry is undertaken. Moreover, the hospital's application last November for incorporation under the Companies Act has been so absurdly misunderstood by the letter-writers alluded to, that we are not surprised that they have been ashamed to sign their names. The meaning of incorporation is, however, an interesting subject which we shall return to shortly. It should be added, of course, that, as Mr. W. Gilmour pointed out, in order for the school to obtain recognition by the dental examining bodies, a mechanical laboratory had to be installed.

### NELSON HOSPITAL, WIMBLEDON, AGAIN.

We understand that Princess Louise, Duchess of Argyle, has consented to open, at a date left to be fixed, the nearly completed Nelson Hospital at Wimbledon, the progress and initiation of which have been recorded fairly often in these columns. It is understood that the council of management, on which there is still a vacancy or two, will probably be strengthened by the addition of some of the most energetic members of the building committee. Another interesting proposal is to increase the medical staff from four to six. After the local practitioners have selected these members, the council of management confirms their election.

### SIR WILLIAM ALLCHIN.

By the death of Sir William Allchin another prominent figure is removed from the medical profession in London, and the Westminster Hospital Medical School loses one of its most brilliant teachers. Sir William Allchin was the author of a popular text-book on medicine, and well known as a genial examiner at the University of London and on the Conjoint Board. As a lecturer on medicine and as Dean of the Westminster School, Sir William Allchin was always a great favourite with the students, both for his sound advice in the latter capacity and for the practical and thorough teaching for which he was noted as a lecturer. Sir William Allchin was originally connected with University College Hospital, from which he qualified in 1869. He was elected a Fellow of the Royal College of Physicians in 1878, and in that body held the offices of Assistant Registrar, Councillor, Censor, Examiner, and Representative on the senate of the University of London. He also served in turn as Harveian Orator, Lumleian and Bradshaw Lecturer as well as Lecturer to the Hunterian Society. Sir William Allchin was Physician Extraordinary to H.M. the King, and Consulting Physician to Westminster Hospital and other institutions. On his retirement from active duty as senior physician at Westminster, the advantages of his great skill as a clinical lecturer were retained by electing him Emeritus Lecturer to the Hospital.

### A BELATED BIOGRAPHY.

Sir William wrote with much self-denial a full life of Sir Andrew Clark, so far as his medical interests and work were concerned. It has not yet been published, because, we understand, the gentleman entrusted with the public portion of Sir Andrew Clark's life has never completed his manuscript. Be this as it may, we hope no time will be lost in publishing this life of Sir Andrew Clark by Sir William Allchin, for it should prove both valuable and interesting. Sir William was requested to write this by Sir Andrew Clark's executors, and was hurried to do it quickly. He therefore pushed on with the work, and must have completed it some fifteen years ago, Sir Andrew Clark having died in 1893.

### THE HOSPITALS AS POPULARISERS OF BOOKS.

A sign of the times—and a very encouraging one —is the steadily increasing interest of all classes of the population in the subject of hygiene. It has long been remarked that in this respect the voluntary hospitals exercise a great influence for good. A sojourn in the wards is bound to impress any patient, even if unconsciously, with ideals of cleanliness, order, discipline, and with the importance there attached to a plentiful supply of fresh air. Another potent influence for the instruction of the masses is a supply of good, cheap, sensible books, dealing with the matter in a comprehensible and interesting way. One of the most recent is Dr. Dingwall Fordyce's *Care of Infants and Young Children* (Edinburgh: Livingstone, 1s. 6d. net). Of this valuable manual we have but one criticism to offer, and that is with respect to certain photographs at the end illustrating the hideous results of meningitis, rickets, hydrocephalus, and other diseases. These would have been far better omitted; but for the rest of the work there can be nothing but ungrudging praise. It is written in a simple, straightforward style, almost without an unnecessary word. Especially is one grateful for the absence of the tone of maudlin, twaddling sentimentalism which so often pervades this sort of book; Dr. Fordyce never speaks of "baby's little legs," for instance.

### THE IRREGULAR SALE OF POISONS.

In spite of much vigilance on the part of the Pharmaceutical Society, and of the imposition of penalties on offenders, frequent irregularities occur in connection with the sale of poisons. It is one of the Society's most important duties to administer the penal clauses of the Pharmacy Acts, and in connection with this work last year 1,244 cases of alleged infringement were investigated, and legal proceedings were taken in 241 cases. As a rule the offenders were unqualified persons who sold poisons which, legally, may only be sold by retail by duly registered pharmacists, and in almost every case in which legal proceedings were instituted the offender was ordered to pay a fine.

### A NEW IDEA FOR NEXT CHRISTMAS.

In December we drew attention to the ingenious device by which the Gravesend Hospital, through its President Lord Darnley, its Chairman Mr. H. D. Stephenson, and its Secretary Mr. W. Pearson, were making Christmas-boxes a source of income to the hospital. In order to help certain other secretaries, who showed anxiety to learn the procedure adopted at Gravesend, we supplement our former statement by a brief analysis of the methods and results of this Christmas-box collection. The hospital area apparently was divided into sixty-five districts, and given into the hands of about the same number of collectors. These succeeded in getting Christmas-boxes to the gross value of £204, which, once the printing bill of £12 had been paid, left approximately £192 to be handed over to the institution. A careful analysis shows that on the average each household would give about one shilling, and each collector bring home rather less than £2 10s.

### THE WORCESTER MEMORIAL TO KING EDWARD.

As we go to press we learn that the first important step towards the realisation of the Worcester memorial to King Edward is being taken by the Mayor of the city, who is to lay the foundation stone of the Infirmary's new wing on Thursday this week. The wing or annexe will contain a laboratory, $x$-ray, and operating rooms, as well as consulting rooms, a surgery, and provision for the nursing staff. Moreover, the new wing has a flat asphalt roof for the use of children. We hope its value will be justified and appreciated, but occasionally, it must be admitted, flat roofs on added wings have presented new difficulties in matters of administration. In the meantime, however, we congratulate the Infirmary on the first stage of its reconstruction work.

### THIS WEEK'S DRUG MARKET.

Business in the drug market remains rather quiet. At the moment cod-liver oil is the article of chief interest. So far the catch of cod and the yield of cod-liver oil have been large, and if the fishing continues as it has begun there is little doubt that we shall see a material reduction in price, although the oil percentage is lower than last year. Should the oil percentage become less, a check would be given to the declining prices. The fact that buyers are holding off indicates that lower prices are anticipated. Essence of lemon and essence of bergamot are again somewhat dearer. Oil of cloves still tends lower in price. Orange peel and valerian root and Indian hemp are dearer and another slight advance in the quotations for sodium salicylate and salicylic acid is not altogether unexpected. Sugar of milk is slightly cheaper and there is also a weaker tendency in crude carbolic acid. Sarsaparilla is rather dearer. Quicksilver is unchanged but an advance in price would not come as a great surprise.

